# The impact of a digital joint school educational programme on post-operative outcomes following lower limb arthroplasty: a retrospective comparative cohort study

**DOI:** 10.1186/s12913-022-07989-1

**Published:** 2022-04-29

**Authors:** Joanne Gray, Stephen McCarthy, Esther Carr, Gerard Danjoux, Rhiannon Hackett, Andrew McCarthy, Peter McMeekin, Natalie Clark, Paul Baker

**Affiliations:** 1 Department of Health and Life Sciences, Northumbria University, Newcastle-upon-Tyne, UK; 2 Department of Anaesthesia, James Cook University Hospital, Middlesbrough, UK; 3 Department of Orthopaedic Surgery, James Cook University Hospital, Middlesbrough, UK

## Abstract

**Background:**

As part of an ongoing service improvement project, a digital ‘joint school’ (DJS) was developed to provide education and support to patients undergoing total hip (THR) and total knee (TKR) replacement surgery. The DJS allowed patients to access personalised care plans and educational resources using web-enabled devices, from being listed for surgery until 12 months post-operation. The aim of this study was to compare a cohort of patients enrolled into the DJS with a cohort of patients from the same NHS trust who received a standard ‘non-digital’ package of education and support in terms of Health-Related Quality of Life (HRQoL), functional outcomes and hospital length of stay (LoS).

**Methods:**

A retrospective comparative cohort study of all patients undergoing primary TKR/THR at a single NHS trust between 1st Jan 2018 and 31st Dec 2019 (*n* = 2406) was undertaken. The DJS was offered to all patients attending the clinics of early adopting surgeons and the remaining surgeons offered their patient’s standard written and verbal information. This allowed comparison between patients that received the DJS (*n* = 595) and those that received standard care (*n* = 1811). For each patient, demographic data, LoS and patient reported outcome measures (EQ-5D-3L, Oxford hip/knee scores (OKS/OHS)) were obtained. Polynomial regressions, adjusting for age, sex, Charlson Comorbidity Index (CCI) and pre-operative OKS/OHS or EQ-5D, were used to compare the outcomes for patients receiving DJS and those receiving standard care.

**Findings:**

Patients that used the DJS had greater improvements in their EQ-5D, and OKS/OHS compared to patients receiving standard care for both TKR and THR (EQ-5D difference: TKR coefficient estimate (*est)* = 0.070 (95%CI 0.004 to 0.135); THR *est* = 0.114 (95%CI 0.061 to 0.166)) and OKS/OHS difference: TKR *est* = 5.016 (95%CI 2.211 to 7.820); THR *est* = 4.106 (95%CI 2.257 to 5.955)). The DJS had a statistically significant reduction on LoS for patients who underwent THR but not TKR.

**Conclusion:**

The use of a DJS was associated with improved functional outcomes when compared to a standard ‘non-digital’ method. The improvements between pre-operative and post-operative outcomes in EQ-5D and OKS/OHS were higher for patients using the DJS. Furthermore, THR patients also had a shorter LoS.

**Supplementary Information:**

The online version contains supplementary material available at 10.1186/s12913-022-07989-1.

## Introduction

The delivery of patient education and support is an essential component of the patient pathway prior to and following any elective surgical procedure [1]. Recent National Institute for Health and Care Excellence (NICE) guidance outlines the importance of shared decision making and describes the key information that should be offered to patients undergoing hip and knee replacement [2]. It stipulates that information should be ‘presented in a format that can be easily understood’ and should start ‘when listed for surgery, then whenever needed throughout their care’ [2]. Further recent multiagency guidance states that pre-operative education should be delivered via a ‘group ‘surgery school’, which may be in-person, via remote access or Hybrid’. The information delivered should include details of what to expect coming into hospital, post discharge recovery and types of complications, principles of pre-habilitation (exercise, nutrition and mental health), alcohol moderation and smoking cessation, and skills development to enable recovery [3].

Group ‘joint (surgery) schools’ are common within orthopaedic arthroplasty surgery pathways [4]. However, despite their popularity and inclusion in national recommendations, there is very little published evidence of their effectiveness in terms of preparing patients for surgery, reducing length of stay and improving patients reported outcome measures (PROMs). In 2017 we sought to develop a joint school program to run alongside our standard pre-assessment pathway for total knee replacement (TKR) and total hip replacement (THR) patients. As part of this process, we chose to create an expanded ‘digital’ joint school (DJS) using an online web-based platform. The content of this platform was aligned with the national guidance produced by NICE and endorsed by the centre for peri-operative care and the Royal College of Surgeons of England [2, 3]. The developed DJS not only provided pre-operative educational ‘joint school’ materials but also delivered a time lined multimedia pathway that supported patients from the point of listing for surgery through to their discharge from secondary care and beyond (pre-habilitation to rehabilitation). It therefore provided a comprehensive system solution across the entire care pathway rather than the limited ‘one off’ educational approach seen with traditional ‘surgery schools’.

We evaluated the impact of the DJS by comparing routinely collected outcome data for a cohort of patients who utilised the DJS with a cohort of patients that receive standard care (‘non-digital’ education and support package). During this period, early adopting surgeons routinely offered the DJS to their patients while the remaining surgeons in the department did not. The aim of this study was to 1) assess whether the DJS affected the observed changes in health-related quality-of-life (HRQoL) and functional outcomes at 6 months following surgery 2) evaluate the impact of the introduction of a DJS upon patient length of stay (LoS) following hip and knee replacement.

## Methods

This is a retrospective comparative cohort study of all patients undergoing primary hip and knee replacement at a single institution between 1st Jan 2018 and 31st Dec 2019. The DJS was introduced alongside standard care in 2017 allowing comparison between those patients that received the DJS and those that did not. The DJS was offered to all patients attending the clinics of early adopting surgeons with no explicit exclusions. The surgeons who were not early adopters continued to offer all of their patient’s standard written and verbal information. Patients who attended the clinics of early adopting surgeons received a written information sheet and verbal offer of registration for the DJS from their surgeon at the point of listing for surgery. The nurse in clinic then subsequently checked with the patient that they had been offered the DJS as they completed their pre-operative paperwork in the clinic after their surgical consultation.

### Intervention

The DJS was developed using the GoWellHealth (GWH) platform [5]. Further information on the development of the DJS is provided in the supplementary material and at https://cpoc.org.uk/case-studies-preoperative-optimisation [3]. Through the platform we created a library of over 100 patient education resources (e.g., information about surgery, exercise videos, lifestyle and wellbeing support, pre-habilitation advice and support, questionnaires to support outcome collection and care after surgery) and support mechanisms (e.g., prompting emails, interactive forms monitoring progress and recovery) in a variety of digital formats (PDF documents, videos, interactive forms, email etc.). The content stored within the library was then combined to create bespoke packages of information (termed a ‘carepac’) that could be delivered over a specific period of time to coincide with key aspects of the care pathway (e.g., listing for surgery, pre-assessment, day of surgery, post-operative surgical recovery, long term rehabilitation) [3]. The creation of the carepacs allowed us to personalise the care delivered to individuals, support different elements of the surgical pathway with a range of complimentary resources and provide a comprehensive package of care that spanned the entirety of the care journey from pre-habilitation to rehabilitation.

Due to the design of the GWH platform all interactions with the DJS were recorded allowing the surgical team to establish the proportion of people offered the DJS that ‘activated’ their GWH account and the level of patient engagement. Activation rates, defined as the patient opening the email inviting them to join the DJS, creating a user password, logging in using their password and accessing at least one piece of the DJS content, was > 80% across the study period. Patients that ‘activated’ their DJS were used as the intervention cohort for this study. Patients that did not ‘activate’ their DJS were included within the comparison group. Patients were consented prior to enrolment into the DJS via their care provider at the point they were listed for surgery. Once activated, the DJS provided a comprehensive package of education and support spanning from surgical listing to 12 months after surgery. Patients who were provided access but did not activate their account were not provided with any further materials, only standard care.

### Comparator

Prior to development of the DJS, patients received a combination of written materials (booklets provided by the hospital orthopaedic team) and verbal information provided by their surgeon at the point of listing for surgery, supplemented by further advice delivered by a specialist arthroplasty nurse or physiotherapist during a pre-assessment appointment 4–6 weeks prior to surgery. THR patients would also be seen by an occupational therapist prior to surgery. This package of care continued to be used by a number of consultants that were not early adopters of the DJS and remained the standard practice for patients not offered the DJS throughout the study period.

### Outcomes

Outcomes and patient level data were collected from routinely collected hospital data sources (Hospital Episode Statistics (HES), National NHS Patient Reported Outcome Measures programme) pre-operatively and 6 months post-surgery for all patients included in the study. The pre- and post-operative questionnaires included a generic preference-based HRQoL measure EQ-5D-3L [6, 7] and condition specific measures of symptoms and disability (Oxford Hip and Knee Scores) (OHS/OKS) [8]. The EQ-5D comprises five questions each assessing a specific dimension of health (mobility, self-care, usual activities, pain, and anxiety and depression) with three response levels (“no problems”, “some or moderate problems”, and “extreme problems”) [6, 7]. Responses are converted into a single score on a scale from 1 (perfect health), 0 (death), to − 0.596 (worse than death with extreme problems in all five dimensions) [6, 7]. The OHS/OKS assesses symptoms and function through 12 items with five response levels. The item scores are summed to generate an overall score that ranges from 0 (worst health status) to 48 (best health status) [8].

### Data sources

Patients were identified from HES data and their associated PROMs record was obtained. The patients were also linked to records held within the DJS platform allowing us to determine who had been registered with the DJS, the patient’s level of compliance and number of interactions they had with the platform between their registration on the DJS at the point they were listed for surgery until 12 months post operation.

The NHS PROMs programme has routinely collected outcome data for all TKR or THRs funded by the NHS since 2009 [9]. All patients are invited to complete a questionnaire immediately before surgery and 6 months after the surgery. PROMs data was linked to HES data within the trust based on a hierarchical deterministic linkage algorithm [9]. The link with HES enabled data acquisition regarding the patient’s sex, age, hospital length of stay after their procedure and The Charlson Comorbidity Index (CCI) [10] and mortality status.

### Missing data

Data regarding age, sex, and hospital length of stay were complete for all patients. One patient had a missing CCI, and two patients had a CCI of − 1 (an implausible CCI score). These patients were excluded to allow for CCI to be used as a covariate for data imputation. Patients with a date of death within 180 days after their date of surgery were assigned a post-operative EQ-5D value of zero.

Pre- and post-operative EQ-5D scores were missing for 864 (35.91%) and 1427 (59.31%) patients respectively with pre-operative OKS/OHS missing for 682 (28.35%) patients and post-operative OKS/OHS missing for 1385 (57.56%) patients. Table 1 below shows the amount of missing data by surgery and care received.

**Table 1:** Missing outcome data by surgery and care

	Total Knee Replacement		Total Hip Replacement	
	Digital Joint School (*n* = 287)	Standard care (*n* = 873)	Digital Joint School (*n* = 308)	Standard care (*n* = 938)
Missing pre-operative EQ-5D	97 (33.80)	304 (34.82)	78 (25.32)	385 (41.04)
Missing post-operative EQ-5D	162 (56.45)	511 (58.53)	156 (50.65)	598 (63.75)
Missing pre-operative OKS/OHS	79 (27.53)	227 (26.00)	62 (20.13)	314 (33.48)
Missing post-operative OKS/OHS	155 (54.01)	480 (54.98)	157 (50.97)	593 (63.22)

All figures are reported as number (percentage). Percentages are given to two decimal places and are percentages of the total number of patients

The nature of the missing data was explored using a series of tests and logistic regressions. While the true relationship between the missing data and the value of variables is unknown, the method used to handle missing data should be based on plausible assumptions about this relationship. The use of an inappropriate method to handle the missing data could lead to misleading results [11]. Results suggested that the data may be missing at random (MAR) or missing not at random (MNAR): the probability that data is missing may or may not be independent of unobserved values [11]. Multiple imputation with predicted mean matching was used to impute missing data as properly specified imputation models can be used to obtain unbiased results [12]. In the analysis, the missingness was assumed to depend on baseline covariates (age, sex, CCI, and by group). Sensitivity analysis was performed to investigate the impact of assuming MNAR and the implication on the results. These analyses were conducted by applying an absolute reduction of the imputed post-operative outcome scores (EQ-5D, OKS/OHS) by 10, 20, 30, 40, 50% iteratively. This was conducted under the assumption that outcome data may be more likely to be missing for patients with worse HRQoL and OKS/OHS [11].

### Statistical analysis

Summaries of patient characteristics were compared between the DJS cohort and those patients receiving the standard care using the appropriate statistical tests (a two-sample t test (for age, CCI, pre-operative EQ-5D value and pre-operative OKS/OHS) or a Chi-Square test (for sex)). The difference between the pre-operative and post-operative patient reported outcomes (EQ-5D, OHS/OKS) for each patient were compared for those patients who used the DJS and those who did not. Linear regression was used to adjust the comparison of differences between pre- and post-operative EQ-5D utility values as well as between pre- and post-operative OHS/OKS for age, sex, CCI and pre-operative scores. Fractional polynomials were used to investigate potential non-linear relationships between the outcome and the factors included in the regression model as continuous variables [13]. A Poisson regression model is often used to investigate count data [14]. However, hospital LoS was found to be over dispersed and, as such, a negative binomial regression model was used instead [13]. This regression adjusted for age, sex, CCI and pre-operative EQ-5D. Potential non-linear relationships using fractional polynomials were also explored.

Regression results are reported with 95% confidence intervals (95% C.I.). All coefficients and reported *p*-values are based on statistical tests adjusted for the specified pre-operative characteristics. *P*-values of less than 0.05 were considered statistically significant. All analyses were undertaken using Stata version 14.2 [15].

### Ethics and approvals

Data for the study was collected as part of a broader service improvement and evaluation project that was registered with the Trust’s research and development department. As part of the consent to enrolment on the DJS each patient also provided consent that their data could be used for audit and research. The strengthening the reporting of observational studies in epidemiology (STROBE) checklist was followed to ensure rigour within this study.

## Results

### Population

Between 1st Jan 2018 and 31st Dec 2019, there were 2406 patients in total, 1160 of which underwent a TKR and 1246 who underwent a THR. For 1160 patients who underwent TKR, 350 (30.17%) were offered the DJS, of which 287 (24.74%) activated it. This was an activation rate of 82%. This resulted in a total of 873 (75.26%) patients who received standard care. For 1246 patients who underwent THR, 371 (29.78%) were offered the DJS, of which 308 (24.72%) activated it. This was an activation rate of 83.02%. This resulted in a total of 938 (75.28%) patients who received standard care.

### Demographic characteristics

For patients who had a TKR or THR, there were key differences in baseline characteristics between those in the DJS group compared those in the standard care group (Table 2). For patients who underwent a TKR, DJS patients were on average younger (mean: 67.72 vs 68.75, *p* = 0.113) and more likely to be male (49.48% vs 42.15%, *p* = 0.03). For patients who underwent a THR, DJS patients were more likely to be younger (mean: 65.70 vs 69.81, *p* < 0.001) and have a lower CCI (mean: 1.92 vs 2.84, *p* = 0.003).

**Table 2:** Demographic characteristics of patients who underwent a TKR or THR by study group

Variable	Total Knee Replacement (***n*** = 1160)			Total Hip Replacement (***n*** = 1246)		
	DJS (***n*** = 287)	Standard care (***n*** = 873)	***P*** value	DJS (***n*** = 308)	Standard care (***n*** = 938)	***P*** value
Age			*p* = 0.113			*p* < 0.001***
Mean (SD^a^)	67.72 (8.51)	68.75 (9.82)		65.70 (10.57)	69.81 (11.29)	
Median (IQR^b^)	69 (63 to 73)	70 (62 to 75)		66.5 (59 to 73)	71 (64 to 78)	
Male			*p* = 0.03*			*p* = 0.373
n (%)	142 (49.48)	368 (42.15)		129 (41.88)	366 (39.02)	
CCI			*p* = 0.182			*p* < 0.001***
Mean (SD)	2.04 (3.50)	2.28 (3.29)		1.92 (3.11)	2.84 (4.48)	
Median (IQR)	0 (0 to 4)	1 (0 to 4)		0 (0 to 3)	1 (0 to 4)	
Pre-operative EQ-5D			*p* = 0.438			*p* = 0.081
n	190	569		230	553	
Mean (SD)	0.36 (0.32)	0.34 (0.33)		0.30 (0.32)	0.26 (0.34)	
Median (IQR)	0.52 (0.55 to 0.69)	0.26 (0.06 to 0.69)		0.19 (−0.02 to 0.62)	0.16 (−0.02 to 0.59)	
Pre-operative OHS/OKS			*p* = 0.634			*p* = 0.077
n	208	646		246	624	
Mean (SD)	17.50 (7.23)	17.20 (8.23)		16.44 (7.89)	15.31 (8.72)	
Median (IQR)	17 (12 to 23)	17 (11 to 23)		15.5 (10 to 21)	15 (9 to 21)	

All figures are to 2 decimal places, *p* values are to 3 decimal places

* *p* value < 0.05

** *p* value < 0.01

*** *p* value < 0.001

^a^Standard Deviation

^b^Inter Quartile Range

The impact of the DJS on the difference between pre-operative and post-operative EQ-5D and OKS/OHS are presented in Table 3. EQ-5D improvements were significantly higher for the DJS group compared to patients receiving standard care for both TKR (*est* = 0.070 (95%CI 0.004 to 0.135) and THR patients (*est* = 0.114 (95%CI 0.061 to 0.166)). Similarly, patients in the DJS group had a significantly greater improvement in their OKS/OHS compared to those in the standard care group. The modelled difference in the OKS improvement was 5.016 points (95%CI 2.211 to 7.820) for TKR patients and the OHS difference was 4.106 (95%CI 2.257 to 5.955) for THR patients.

**Table 3:** Association of DJS use on outcomes changes for patients who underwent TKR or THR

Measurement	Surgery	Variables	Est^a^. (SD)	p value	95% C.I.
Change in EQ-5D	Patients who underwent a TKR	Sex	−0.031 (0.024)	0.200	−0.079 to 0.017
		Age	0.003 (0.001)	0.026*	0.000 to 0.006
		CCI	−0.004 (0.004)	0.315	−0.014 to 0.004
		Pre-operative EQ-5D	−0.259 (0.037)	< 0.001***	− 0.333 to − 0.186
		DJS	0.070 (0.032)	0.039*	0.004 to 0.135
	Patients who underwent a THR	Sex	0.048 (0.027)	0.075	−0.005 to 0.102
		(Age/10)	0.253 (0.086)	0.005**	0.080 to 0.427
		(Age/10)^2^	−0.020 (0.007)	0.005**	−0.033 to − 0.006
		CCI	−0.016 (0.003)	< 0.001***	−0.022 to − 0.010
		Pre-operative EQ-5D	−0.337 (0.039)	< 0.001***	−0.416 to − 0.258
		DJS	0.114 (0.026)	< 0.001***	0.061 to 0.166
Change in OKS/OHS	Patients who underwent a TKR	Sex	−1.651 (1.293)	0.202	−4.189 to 0.887
		Age	0.188 (0.067)	0.005**	0.056 to 0.321
		CCI	−0.097 (0.188)	0.606	− 0.466 to 0.272
		Pre-operative OKS	−0.062 (0.096)	0.519	−0.255 to 0.130
		DJS	5.016 (1.429)	< 0.001***	2.211 to 7.820
	Patients who underwent a THR	Sex	1.104 (0.990)	0.271	−0.891 to 3.099
		Age/10	4.152 (1.791)	0.025*	0.540 to 7.764
		(Age/10)^3^	−0.033 (0.014)	0.021*	−0.062 to − 0.005
		((CCI + 1)/10)^−2^	0.040 (0.009)	< 0.001***	0.022 to 0.057
		Pre-operative OHS	−0.179 (0.056)	0.002**	−0.291 to − 0.067
		DJS	4.106 (0.937)	< 0.001***	2.257 to 5.955

All figures are to 3 decimal places

^a^Coefficient estimate (*est*)

* *p* value < 0.05

** *p* value < 0.01

*** *p* value < 0.001

The impact of the DJS on hospital LoS is presented in Table 4. The hospital LoS was significantly lower for those who underwent THR and were in the DJS group (Incidence Rate Ratio (IRR) *est* = 0.667 (95%CI 0.585 to 0.760)) compared to those receiving standard care; those in the DJS group had a LoS 33.3% lower than the comparator group, when all other variables were adjusted for within the models. There was no observed effect on LoS of the DJS in patients that underwent TKR.

**Table 4:** Association between DJS use and LoS for patients who underwent TKR or THR

Surgery	Variables	IRR *est*.^a^ (SD)	p value	95% C.I.
Patients who underwent a TKR	Sex	0.962 (0.042)	0.382	0.879 to 1.053
	Age/10	0.293 (0.067)	< 0.001***	0.183 to 0.470
	(Age/10)^2^	1.110 (0.019)	< 0.001***	1.072 to 1.150
	ln((CCI + 1)/10)	1.210 (0.030)	< 0.001***	1.149 to 1.274
	Pre-operative EQ-5D	0.810 (0.072)	0.027*	0.673 to 0.974
	DJS	1.042 (0.051)	0.407	0.942 to 1.154
Patients who underwent a THR	Sex	0.858 (0.044)	0.010**	0.768 to 0.957
	(Age/10)^2^	0.932 (0.014)	< 0.001***	0.902 to 0.962
	(Age/10)^3^	1.008 (0.015)	< 0.001***	1.005 to 1.012
	CCI	1.051 (0.006)	< 0.001***	1.038 to 1.065
	Pre-operative EQ-5D	0.854 (0.115)	0.265	0.638 to 1.144
	DJS	0.667 (0.177)	< 0.001***	0.585 to 0.760

All figures are to 3 decimal places

* *p* value < 0.05

** *p* value < 0.01

*** *p* value < 0.001

^a^Incidence Rate Ratio estimate (IRR *est*.)

Analysis of the imputed post-operative outcome scores suggested that, for all outcomes for both TKR and THR patients, with the exception of EQ-5D for the TKR patients, any absolute reduction in the imputed post-operative values had no effect on the statistical significance of the DJS. Furthermore, for TKR patients, reductions in the imputed post-operative EQ-5D of up to 30% still resulted in the DJS being statistically significant.

## Discussion

The present study found that, for patients undergoing THR or TKR, use of a DJS produced significant improvements in patient reported outcomes measures (OHS/OKS) and HRQoL (EQ-5D) compared to patients that do not use the platform. The DJS was also associated with a reduction in hospital LoS following THR surgery.

Data collected by the national PROMs program linked to information from the National Joint Registry for England, Wales, Northern Ireland, and the Isle of Man reports an average OKS/OHS PROMs improvement after surgery of 17.1 (OKS) and 22.0 (OHS) [16]. Reported improvements in the EQ-5D are 0.33 (TKR) and 0.45 (THR) [16]. The improvements seen with our DJS were better than these national figures (OKS 20.6, OHS 24.1, EQ-5D TKR 0.42, EQ-5D THR 0.52) with an unadjusted size effect difference of 2–3 points for the OKS/OHS and 0.07–0.09 points for the EQ-5D. After adjustment for variation in group demographics these size effect difference increased further (4–5 points for the OKS/OHS and 0.07–0.11 for the EQ-5D). Size effects of this magnitude are greater than the published minimally clinically important differences for these scores [17–19] and larger than the size effects seen with discrete surgical interventions in TKR/THR patients [20–23]. This highlights the potential value of approaches that span the peri-operative pathway rather than just one aspect of care.

NHS England has recently implemented a Reducing Length of Stay programme which aims to expedite discharge, avoid discharge delays, and minimise the risks associated with prolonged hospital stays [24]. The programme’s five key principles include: planning discharge from the beginning, sharing discharge decision making, establishing systems for frailer patients, involving multidisciplinary teams, and encouraging a ‘home first’ approach [24]. In-person pre-operative educational programs have been shown to significantly reduce LoS for THR/TKR patients [25]. However, our experience delivering a DJS aligned to these principles, demonstrates that a digital solution can also produce significant reductions in LoS for TKR/THR patients. Furthermore, our DJS supports the ‘home first’ and ‘care closer to home’ principles that are integral to the NHS’ long term vision for care delivery [26].

Our DJS aligns with the most recent NICE guidelines for joint replacements and patient experience [2, 27]. In providing patients with specific information regarding their planned surgery, it encourages patients to become actively involved in their care, promoting self-management of their health both before and after surgery, ensuring maximal outcomes. The benefits of optimising information sharing and the inclusion of patients in decision making are relevant to all healthcare interactions across all healthcare settings [28]. As such, the impact we have been able to demonstrate using a digital approach to patient education and support is likely to be realised in other healthcare settings in which a digital solution for information sharing, shared decision making, and patient support are appropriate. Our results are most generalisable to elective care pathways in surgical care where a digital program could be adopted and implemented using a similar approach [29]. The ability to duplicate key information across multiple pathways (e.g. venous thromboembolic risks, pain management, information about anaesthesia etc) for multiple conditions also brings an economy of scale meaning subsequent ‘digital surgery schools’ can be rapidly adapted and mobilised simply by the addition of procedure specific information within an established framework of ‘generic’ surgical information. Qualitative work sampling from patients using the DJS demonstrated that patients on the platform felt they had greater control of their own health behaviours and that engagement with the DJS had contributed to their recovery after surgery [30]. Patients spoke positively about the value of external email prompts to keep them engaged, the ability to invite carers, family, and friends onto their DJS program to enable social support and awareness of what they were going through, and the importance of a structured program spanning the entire care episode [30]. Patients often feel overwhelmed by the volume of information delivered in a ‘one off’ face to face setting [30] and a structured digital solution allows patients to view information across a period of time, at a time that is convenient for them and revisit digital content when clarification is needed. We believe it is these factors that have produced the statistically significant findings observed in our analyses and, because these benefits are not limited to the arthroplasty population, similar improvements may be observed if a similar digital solution was utilised for other surgical procedures and medical conditions.

A range of formats for the delivery of patient education have been reported previously [31, 32]. These include ‘in person’ education classes, web-based programs, and audio-visual resources (such as video and educational booklets) [31, 32]. Patients attending ‘in person’ education classes report a number of benefits, including improved quality of life following surgery [32]. However, it is acknowledged that the success of these classes relies on the patients attending the sessions. The benefit of the DJS is that the information, in a variety of formats, can be accessed at any time of the day from a variety of digitally enabled devices (computer, tablet, phone) and has all of the content available in a single online library [5].

The results of our study suggests the DJS is associated with reduced LoS for THR by an average of 33.3%. In 2018–19 (prior to Covid-19), there was an estimated 81,130 THRs in the UK NHS [9], with a typical LoS of 3 to 5 days [33]. Given the average cost of a bed day in the NHS is £416.90 [34, 35], assuming a conservative LoS for THR (3 days), the adoption and implementation of a nationwide DJS program has the potential to reduce costs by approximately £34 million, assuming 100% adherence.

A potential limitation of this study is that the patients included in the DJS group were those that had activated their accounts and accessed at least one piece of content. We did not examine the relationship between intensity of use and outcomes. Another potential limitation is that confounding adjustment was dealt with by the traditional outcome regression model. However, it is essential that this incorporates a perfectly specified model to reduce residual confounding and resulting bias. Furthermore, propensity scoring is an increasingly popular method of controlling potential confounding in observational studies that compare the effectiveness of healthcare interventions [36, 37]. It is typically used in retrospective cohort studies such as this and involve fitting regression models to predict treatment groups based on selected characteristics derived from administrative healthcare data or electronic health record data [37]. However, previous research suggesting the equivalence of confounding adjustment to various propensity score-based approaches [38, 39]. Our retrospective study used HES data that were collected in the past for another objective. With a lack control over data collection, it is possible that inaccuracies in the data are present which results in a source of information bias. Another limitation of this study is that the DJS was still in its infancy in 2018 and 2019 and has since undergone multiple iterations to improve patient uptake and engagement We may therefore be underestimating the relationship between it and health outcomes. Finally, there was large amounts of missing data for both pre- and post-operative outcome scores. However, multiple imputation with predictive mean matching was used to account for this, with sensitivity analysis being run to ensure the robustness of the results [12].

TKR and THR are common procedures in the NHS [9] and are both an effective intervention to improve patient’s HRQoL and a cost burden to the NHS. Our analysis shows that use of a DJS can further improve health related quality of life for patients who undergo a TKR or a THR. In addition to this, there is also evidence that the use of a DJS may also reduce the length of stay in hospital for those who undergo THR and thereby reduce the costs to the NHS. In light of the results of this study, further research into the impact of a DJS on patients undergoing TKR and THR in the form of a randomised control trial could provide further high-quality evidence on this issue.

## Supplementary Information

**Additional file 1.**

**Additional file 2.**

## Data Availability

All data generated or analysed during this study are included in this published article as part of its supplementary information files.

## Abbreviations

DJS: Digital ‘Joint School’
TKR: Total Knee Replacement
THR: Total Hip Replacement
CCI: Charlson Comorbidity Index
HRQoL: Health-Related Quality of Life
OHS: Oxford Hip Score
OKS: Oxford Knee Score
LoS: Hospital length of stay
HES: Hospital Episode Statistics
PROMs: National NHS Patient Reported Outcome Measures
GWH: GoWellHealth platform
MAR: Missing at random
MNAR: Missing not at random
IRR: Incidence Rate Ratio
STROBE: Strengthening the reporting of observational studies in epidemiology
est.: Estimate
NICE: National Institute for Health and Care Excellence
GIRFT: Getting It Right First Time
SD: Standard Deviation
IQR: Inter-quartile Range
95% C.I: 95% Confidence Interval
