# Supplementary material for: The impact of a digital joint school educational programme on post-operative outcomes following lower limb arthroplasty: a retrospective comparative cohort study
Source: BMC Health Serv Res. 2022 Apr 29;22:580. doi: 10.1186/s12913-022-07989-1 (PMC9053557; doi:10.1186/s12913-022-07989-1)
Supplement: Supplementary file 1 — Additional file 1. [file 12913_2022_7989_MOESM1_ESM.docx]

**Supplementary Information relating to the DJS**

Further information relating to the DJS.

This information is copied from sections of our case study which is freely available on the case studies section of the Centre for Peri-operative Care website https://cpoc.org.uk/case-studies-preoperative-optimisation (Development and Impact of a ‘digital joint school’ within an elective orthopaedic surgery pathway (South Tees Hospitals NHS Foundation Trust))

Development of the DJS

In 2017 we needed to redesign our surgical preparation pathway and patient facing educational materials. We wanted to extend the joint school approach using a digital platform that could support decision making and provide education and support across the entirety of the care pathway. This led us to develop a bespoke digital patient and education support program to cover all aspects of care, starting at surgical listing and continuing beyond discharge from the orthopaedic service.

Work done locally at South Tees Hospitals NHS Foundation Trust showed that there was significant variation in the type, quantity and detail of information requested by and delivered to patients during their perioperative care episode (Norman 2015 MSc thesis). Some patients wanted minimal information about the processes of care whereas other request detailed information about a range of clinical issues. A ‘one size fits all’ approach to information content and delivery was failing to address the needs of all patients.

Furthermore, a review of the orthopaedic care pathway for hip and knee replacement patients demonstrated a number of issues in the way patients received education and support.

1. There was a wide variety of staff involved in the delivery of patient education and support including physiotherapists, occupational therapists, nurses, nurse practitioners. The variation in team members had resulted in delivery of patient education material that differed in its content and often lacked key information.
2. The timing of the delivery of patient education and support was inconsistent. Sometimes this happened only a few days before surgery giving patients little time to prepare themselves and their home environment.
3. Rapid turnover over of trainee doctors on the ward, many of whom had no prior orthopaedic experience, meant that many felt uncomfortable delivering advice and support to arthroplasty patients during their stay. This led to inconsistent information at the point of discharge from hospital.
4. There was significant duplication of work between team members and a limited access to key information as they maintained their own paper records which weren’t always available in the patients’ medical records.
5. There was pressure on clinic space leading to our pre-admission service being relocated to a local community hospital. While this had some advantages, it led to a lack of oversight of the process for individual patients by their treating consultants.
6. This approach led to a large number of patients contacting the ward and secretarial services with queries about upcoming operations. This was a significant burden of work for the ward and secretarial teams.

Having identified the aforementioned problems the Trust looked for solutions. It was felt a ‘digital joint school’ approach might provide the most appropriate way forward. We therefore developed a solution in collaboration with GoWellHealth (GWH) (https://gowellhealth.co.uk/about-us) who had a web-based healthcare platform that allowed creation of bespoke patient education and support programs.

In developing a solution alongside GWH, a list of desirable requirements were drawn up:

- Clear and consistent delivery of patient information that can be delivered in a variety of formats (written, video, email, interactive forms etc).
- Ability to track patient engagement and monitor patient use.
- To have the capability to collect information from patients including the collection of Patient Reported Outcome Measures (PROMs) and health and social care information
- It should be clinician driven and controlled, the hospital orthopaedic team should be in charge of what their patients see and the information they receive.
- Transparent information delivery – creation of a program of information that is visible to all members of the hospital orthopaedic team with information about individual patients being available to all registered healthcare team members at all times.
- Adaptability – Ability to change and update programs and information in response to patient demand and need.
- Ability to include all members of the hospital orthopaedic team to increase engagement and buy-in with the process.
- 2-way communication with patients (patient can contact surgical team and surgical team can contact patient)
- Secure portal for patient communication and electronic correspondence (safe mechanism to send patient letters out negating the need for postage and its associated cost).
- Ability to contact a cohort of patients in one action. For example a blanket email or document broadcasted to all patients simultaneously which means administrative staff do not need to contact patients individually
- Data ownership – South Tees Hospitals NHS Foundation Trustown the data about their patients and have confidence that this information will not be sold to a third party
- Ability to use in all orthopaedic sub-specialities (i.e. not limited to elective hip and knee replacement) and to be able to expand across the organisation to other speciailities (e.g. thoracics) if desired.

GWH is a web-based platform, this means that patients are not limited to an app-based program and the information can be accessed using a variety of devices such as phones, tablets and laptops.

Patients are registered to GWH via their care provider at the point of listing for surgery to a bespoke digital clinic (e.g. our orthopaedic patients were registered to the Orthopaedic clinic). This allows multiple ‘clinics’ to be set up within the same platform to support the management of patients in other specialities.

Patient education resources and support mechanisms (e.g. prompting emails, interactive forms monitoring progress and recovery) were created within the GWH platform using a variety of digital formats (PDF documents, videos, interactive forms, email etc.). Each piece of individual content is uploaded and stored in a library of content. The content within the library can then be combined to create a targeted ‘carepac’ of structured information over a particular period of time. For example 10 pieces of individual content including PDF documents, videos and interactive forms can be combined to create a ‘carepac’ delivered over a 3 week period to target leg conditioning and exercises prior to surgery; or a more comprehensive carepac involving >100 pieces of content developed by the wider hospital orthopaedic team can be combined and structured in the months before surgery to provide a pre-operative ‘digital joint school’ covering all aspects of care. Our patients receive multiple carepacs targeted to their needs and timed to key events within their care pathway (listing for surgery, pre-assessment, hospital inpatient stay, post-operative rehabilitation)

The advantage of being able to structure the content within the carepac is that the care team have control over the timing the content is delivered to the patient and the way in which this is delivered. Additionally, it does not overwhelm the patient by delivering the information all at once, instead allowing for chunking and repetition of information, all of which are proven techniques to help increase patient engagement and emphasising key information. Content can easily be created, uploaded, edited or deactivated, allowing for adaptability in the Trust’s response to patient demand and need.

Additional features created within GWH included:

- Interactive forms – allowing data collection from patients e.g. PROMs and health screening questionnaires that allow potential health problems to be recognised early in the patient pathway.
- Communication module – 2 way client communication, allowing patient to contact their care team within the secure platform via a central route. This allowed central co-ordination of patient queries and ensured they could be triaged and directed effectively. For example using the GWH platform our team acknowledged 93.3% of communications received through the plaform within 24 hours, with 66.3% of these also being resolved by the orthopaedic care team within this timeframe. Over half (56.9%) of communications were resolved via the GWH communication module and anoter 29.5% by an additional orthopaedic telephone consultation
- Broadcast – This allows pieces of content to be selected and sent out to groups of individuals in one action.
- Reports – Detailed reports of patient engagement and activity on the platform can be pulled.
- Using this information, bespoke reports can be created for individual patients.
- Health Professional access – All healthcare team members (surgeons, nurses, physiotherapists etc.) can have access to the platform via their own login, viewing the content and information provided to each and every patient within the clinic.

The following barriers were encountered:

1. Information governance: We required each patient’s personal details and email to register them on GWH. This initially required us to take written consent to use this data for this purpose to satisfy local information governance requirement. Through the COVID pandemic written consent became impractical and we moved to a verbal consent process supported by an email explanation of data use and handling with an option to opt out should patients request their data not be used.
2. Staff engagement: We encountered early issues with clinic staff not offering and encouraging uptake following listing for surgery. This required repeated staff education and training.
3. Funding: It was initially difficult to secure funding for a digital approach. This required close collaboration with the GWH team to demonstrate benefits and create a business case for the technology.
4. Cultural change: Moving from an analogue to digital approach was met with resistance by some team members
5. Inequalities: Some people are not digitally enabled (lack of internet and no email). The program is designed so that a patient can be registered to a family member or carers email address. It encourages families and carers to become involve in the patients care. Despite this a small percentage of people (approximately 10-20%) could not be registered on the GWH platform.

The following enablers were encountered:

1. There was a ‘need for change’ rather than change for changes sake. There was recognition within the team of the benefits of a digital approach.
2. The team had the support of colleagues to do something different. A number of colleagues were strong advocates and early adopters of the approach and helped to bring others along with them.
3. We had strong support from the GWH team. This included help developing our pathway and the content within it and suggesting improvements based on their experience in other healthcare settings.
4. After the initial phase of work we were able to gain funding for administrative support for the program. This enabled more timely registration of patients, more responsive communications and frequent review of the content of our pathways.
5. Interest from other clinical teams in the Trust helped to ‘raise the profile’ of our work and enabled the creation of an integrated digital solution to a number of clinical pathways within the GWH platform. This has lead to the development of a pre-habilitation program for orthopaedic patients housed within the platform that can be seamlessly offered to all patients within our digital joint school.

The pieces of content have been designed by a range of team members involved in elective hip and knee joint replacements, including:

• Surgeons

• Surgical care practitioners

• Nurses

• Physiotherapists

• Occupational therapists

• Anaesthetists

• Ward teams

The digital joint school was developed by the lower limb arthroplasty team within the orthopaedic department at South Tees Hospitals NHS Foundation Trust.

We regularly engage with our patients to improve the design and content of the program. We have done this in a number of ways:

- Patient interviews

- Patient satisfaction surveys

- Patient experience surveys

- Assessment of patients confidence with digital technology

- Analysis of patient communications within the program

- Patients rate all content using a ‘smiley face tool. This provides real time feedback about the usefulness of content and acts as a driver for change within the program

Work is also ongoing with the ‘Arthroplasty 4 lower Limb’ PPI group to develop research questions related to this work.

Our program links to the NHS long term plan. With the digital transformation plan the NHS describes the need to ‘improve how the NHS delivers its services in a new and modern way; providing faster, safer and more convenient care.’ Our program aligns with this approach.

The Department of Health’s Five Year Forward View has encouraged efforts to deliver more healthcare out of acute hospitals and closer to home, with the aim of providing better care for patients. Our program provides high quality care and support in the home, reducing the requirement for patients to travel to face to face appointments.

Our program is also aligned with NICE clinical guidelines for the patient population (NG157) and the principles of the Getting It Right First Time (GIRFT) initiative.

We have worked with other clinical teams within secondary care to develop complimentary care pathways. The intention is to create a number of interrelated care pathways that can be delivered seamlessly in one digital platform. This will maximise patient care and experience and can be individualised to the patients care needs, providing truly bespoke care. We are currently working alongside our anaesthetic and Public Health (South Tees) colleagues to integrate their community-based pre-habilitation service (PREPWELL - https://www.southtees.nhs.uk/services/prepwell-project/) within the GWH platform.

We have also expanded our approach to other clinical areas within orthopaedics e.g., Hand surgery, Foot and Ankle Surgery, Trauma; and to other surgical specialities e.g. Thoracic surgery.

To ensure robust evaluation of our outcome measures, and future scalability, we have also partnered with the Health Economics team from Northumbria University.
